# Thyroid Cancer: Focus on Invasion and Metastasis Mechanisms, Therapeutic Target and Drug Treatment

**DOI:** 10.3390/cancers15194762

**Published:** 2023-09-28

**Authors:** Vasyl Vasko

**Affiliations:** Department of Pediatrics, Uniformed Services University of the Health Sciences, 4301 Jones Bridge Rd, Bethesda, MD 20814, USA; vasyl.vasko.ctr@usuhs.edu

Understanding the molecular processes driving thyroid cancer invasion, metastasis, and resistance to therapy is essential for the advancement of novel treatment approaches. This Special Issue “Thyroid Cancer: Focus on Invasion and Metastasis Mechanisms, Therapeutic Targets, and Drug Treatment”, contains a collection of studies that encompass various scientific findings addressing different aspects of thyroid cancer biology. Additionally, it addresses emerging questions in thyroid cancer diagnosis and the responses of thyroid cancer to treatment.

Thyroid nodules are common, with approximately 50% of individuals aged 60 having an enlarged thyroid gland. Most of these nodules are benign, with only 7–15% being thyroid cancer. Diagnosing benign follicular thyroid adenoma (FTA) and follicular thyroid cancer (FTC) can be challenging, and it remains unclear whether FTC and FTA share a common or distinct genomic background. In a study conducted by Borowczyk et al. [1], the authors analyzed a series of FTA and FTC using comprehensive microarray analysis with the goal of identifying recurrent regions of loss of heterozygosity (LOH). The total number of LOH events was found to be higher in FTC compared to FTA. The most common LOH event occurred at 16p12.1, which encompasses many cancer-related genes, including TP53. This LOH was detected with similar frequencies in samples from both FTA and FTC patients. Alterations in 11p11.2–p11.12 were detected only in FTA, while 12q24.11–q24.13 were more often observed in FTC than in FTA. In summary, the results of this study suggest that FTA and FTC may share a common genetic background. Additionally, this study proposes that 12q24.11 LOH may serve as a potential marker of malignancy.

Papillary thyroid carcinomas (PTC) are typically associated with a good prognosis; however, certain variants of PTC may exhibit more aggressive behavior. The tall cell variant (TCV) of papillary thyroid carcinoma (PTC) is characterized by cancer cells with a height at least three times their width. TCV usually manifests in older individuals, presents with a larger size, and displays more extra-thyroidal extension and metastases compared to classical PTC. Proietti et al. [2] conducted a retrospective analysis of 989 TCV patients with a median follow-up of 5.6 years. They examined the recurrence-free survival (RFS) and distant recurrence-free survival (DRFS) rates in relation to the patients’ age. Additionally, for a subgroup of patients with available follow-up data, the mutational status of BRAF (exon 15) and the TERT promoter were investigated. This study demonstrates that TCV is not exclusively found among older patients. There were no significant differences in the mutational status of BRAF among different age groups, whereas mutations in the TERT promoter were exclusively present in older patients. The results of this series indicate that TCV morphology alone in PTCs does not carry the same negative prognostic significance in the younger population as it does in the older population.

Treatment modalities for thyroid cancers include surgery, radioiodine (RAI) therapy, and thyrotropin (TSH) suppressive therapy. These therapeutic options have variable effectiveness, and research on mechanisms underlying the response to treatment is warranted. Autoimmune lymphocytic thyroiditis (AIT) may be associated with the development of papillary thyroid cancer (PTC), and previous studies have shown that AIT may impair radioiodine (131I) uptake. Gheorghe et al. [3] examined the effects of RAI treatment on anti-thyroglobulin antibodies, matrix metalloproteinase-9, TIMP-1, as well as TNF-alpha and its receptors TNFR1 and TNFR2 in patients with PTC and concomitant AIT. The results of this study demonstrated that the mean radioactivity of blood samples collected after 131I intake was higher in the PTC + AIT group than in PTC. In the PTC group, 131I therapy modulates the production of cytokines in situ, increasing the antitumor immune response. On the contrary, in the presence of chronic inflammation due to AIT, 131I therapy amplifies innate immunity, leading to a weaker development of adaptive immunity.

In the management of patients with differentiated thyroid cancer, measurements of serum thyroglobulin (Tg) provide important information about the presence of residual, recurrent, or metastatic disease. Szujo et al. [4] evaluated the diagnostic and prognostic roles of postoperative assessment of stimulated and one-year postablative non-stimulated Tg. The individual lowest and highest non-stimulated Tg values during the entire follow-up were also assessed. This study demonstrated that the detection of non-stimulated Tg had excellent diagnostic accuracy in predicting structural disease. The risk classification based on these data was significantly more accurate regarding outcome than that based on the detection of postoperative stimulated Tg. The results of this study suggested that a patient’s risk category can be revised based on a single Tg measurement.

Anaplastic thyroid carcinoma (ATC) is a highly aggressive thyroid tumor with a poor prognosis. There are limited choices for the effective treatment of this type of thyroid cancer. Despite numerous attempts to explore new therapeutic targets associated with ATC’s prognosis, no effective treatment has been reported to date. Toda et al. [5] have examined the potential utility of antibody–drug conjugates (ADCs) for the treatment of ATC. ADCs are compound-binding antibodies with cytotoxic low-molecular-weight drugs with chemical linkers. ADCs bind to the antigen on the cell membrane surface and are internalized by endocytosis. The linker is then cleaved and releases the drug into the target tumor cells. Authors examined the expression of the ADC targets using the tissue microarrays that were constructed from 54 ATCs. Samples demonstrated that glycoprotein non-metastatic B and B7-H3 were expressed in most anaplastic thyroid carcinoma tissues. Trophoblast cell surface antigen 2 and nectin-4 were expressed in 65% and 59% of anaplastic thyroid carcinoma tissues, respectively. Trophoblast cell surface antigen 2 was highly expressed in ATC undifferentiated from PTC. The results of this study suggested that ADC targeting these cell membrane proteins could be a potential therapeutic strategy for ATC treatment.

There is an epidemiologic link between obesity, insulin resistance, diabetes, and certain cancers. The prevalence of obesity and diabetes is on the rise, and additional epidemiological data suggest a potential connection between obesity and the risk of thyroid abnormalities. Factors that may link obesity and diabetes to thyroid proliferative disorders include elevated levels of circulating insulin, increased body fat, high blood sugar levels, and the use of exogenous insulin. However, the mechanisms underlying the associations between obesity, diabetes, and thyroid proliferative disorders are not yet fully understood. The review manuscript by Kushchayeva et al. [6] summarizes the current evidence regarding the mechanisms and epidemiological associations of obesity, insulin resistance, and the use of anti-diabetes medications with benign and malignant proliferative disorders of the thyroid. The authors analyze complex relationships involving obesity, chronic inflammation, hyperglycemia, hyperinsulinemia, genetic predisposition, crosstalk between proliferative pathways, and various anti-diabetes medications, which may have potential but unknown off-target effects that could affect the risk of thyroid cancer.

Calcium signaling plays a crucial role in numerous cellular processes, including the regulation of muscle contraction, cell proliferation, mitochondrial function, and the regulation of cell membrane potential. Therefore, the actions of calcium signaling hold significant physiological importance for the normal functioning of cells. However, many processes regulated by calcium, including cell movement and proliferation, are also pertinent to cancer. In their review, Asghar et al. [7] summarize information on the role of calcium signaling in normal thyroid cells and its involvement in thyroid cancer. Data indicating the expression of ion channels from the TRP family of ion channels, as well as STIM- and Orai proteins, suggest that calcium signaling may play a crucial role in the development of thyroid cancer. The authors also discuss the potential use of nanoparticles carrying methotrexate for the treatment of thyroid cancer.

The collection of manuscripts in the special issue addresses various aspects of thyroid cancer biology, along with innovative approaches to thyroid cancer diagnosis and treatment. This collection has expanded our current knowledge of the molecular mechanisms underlying thyroid cancers and provides additional insights into diagnostic efficacy, risk assessment, and potential new treatment options for patients with thyroid cancer.
